# Pd-containing magnetic periodic mesoporous organosilica nanocomposite as an efficient and highly recoverable catalyst

**DOI:** 10.1038/s41598-022-11918-x

**Published:** 2022-05-13

**Authors:** Maryam Neysi, Dawood Elhamifar

**Affiliations:** Department of Chemistry, Yasouj University, Yasouj, 75918-74831 Iran

**Keywords:** Catalyst synthesis, Heterogeneous catalysis

## Abstract

A novel magnetic ionic liquid based periodic mesoporous organosilica supported palladium (Fe_3_O_4_@SiO_2_@IL-PMO/Pd) nanocomposite is synthesized, characterized and its catalytic performance is investigated in the Heck reaction. The Fe_3_O_4_@SiO_2_@IL-PMO/Pd nanocatalyst was characterized using FT-IR, PXRD, SEM, TEM, VSM, TG, nitrogen-sorption and EDX analyses. This nanocomposite was effectively employed as catalyst in the Heck reaction to give corresponding arylalkenes in high yield. The recovery test was performed to study the catalyst stability and durability under applied conditions.

## Introduction

In recent years, magnetite nanoparticles, due to their magnetic properties, biocompatibility and easy separation have received a great deal of attention in various fields of science and technology. These have a lot of applications in the areas of catalysis, sensors, drug delivery, water purification and separation^1–6^. However, if the surface of these NPs is left untreated, they oxidize easily and large clusters are formed by the agglomeration of small Fe_3_O_4_ NPs that limit their use for practical applications. To overcome these problems, various shell/covers such as noble metals, metal oxide, silica and polymers have been employed for the protection of magnetite NPs^5,7–14^. Among these, silica shells due to their high chemical stability, versatility for surface modification and great biocompatibility are known to be one of the most suitable coating layers. Hence, silica-coated MNPs provide a vast perspective for designing efficient magnetic catalyst supports^14–19^. On the other hand, periodic mesoporous organosilica (PMOs) are a desirable class of organic–inorganic materials that have emerged as an ideal shell for MNPs, due to their excellent properties such as high surface area, high lipophilicity and high thermal and mechanical stability^20–23^. Some recently reported magnetic catalytic systems include Fe_3_O_4_@SiO_2_/Pr-*N* = Mo[Mo_5_O_18_]^24^, Fe_3_O_4_@SiO_2_@HPG-OPPh_2_-PNP^25^, Fe_3_O_4_@SiO_2_^14^, Fe_3_O_4_@SiO_2_/Shiff-base/M^26^, Fe_3_O_4_@nSiO_2_@mSiO_2_^27^ and Fe_3_O_4_@SiO_2_@TiO_2_^28^.

Ionic liquids (ILs), due to their ability to dissolve a diversity of compounds, have attracted tremendous attention in chemistry and material sciences in the last decade^29–31^. In particular, recently imidazolium-based ILs have been widely used as an outstanding stabilizer for metal nanoparticles during catalytic reactions; also, as a linker that connects catalyst to solid-supports which further enhance the catalytic activity^32–34^. Some of newly developed systems are, Fe_3_O_4_/KCC-1/IL/HPW^35^. Fe_3_O_4_@SiO_2_@(CH_2_)_3_-imidazole-SO_3_H^36^, L-proline-IL-SiO_2_@Fe_3_O_4_^34^, Fe_3_O_4_@nSiO_2_@mSiO_2_/Pr-Imi-NH_2_.Ag^33^ and Fe_3_O_4_@SiO_2_@MIPs^32^.

The Heck coupling reaction is one of the most important organic reaction involving Pd-catalyzed coupling of aryl halides and olefins in the presence of a base. Some of the magnetic catalysts that have been used for the Heck reaction are Fe_3_O_4_@DAG/Pd^37^, Fe_3_O_4_@SiO_2_@Carbapalladacycle^38^, Fe_3_O_4_@SiO_2_–imid–PMA^39^, Fe_3_O_4_@PAA-Pd(II)^40^ and Pd/-AlOOH@Fe_3_O_4_^41^.

In view of the above, in this study, a novel Pd-containing magnetic IL-based PMO (Fe_3_O_4_@SiO_2_@IL-PMO/Pd) is prepared, characterized and its catalytic application is investigated in the Heck reaction.

## Experimental section

### Preparation of Fe_3_O_4_@SiO_2_@IL-PMO nanoparticles

First, the Fe_3_O_4_@SiO_2_ NPs were prepared according to a previous report^42^. In order to prepare Fe_3_O_4_@SiO_2_@IL-PMO, 0.5 g of Fe_3_O_4_@SiO_2_ was added to a flask containing distilled water (5 mL), HCl (2 M, 11 mL) and KCl (3 g) while stirring at 40 °C. Then, 1.5 g of pluronic P123 was added and stirring was continued at 40 °C for 3 h. Next, 0.2 g of 1,3-bis(trimethoxysilylpropyl)imidazolium chloride and 1.5 mL of tetramethoxysilane (TMOS) were added and the resulted mixture was stirred at 25 °C for 24 h under an argon atmosphere. The resulted combination was aged for 72 h at 100 °C. After that, the product was separated using an external magnet, washed with water and EtOH and dried at 70 °C for 12 h^43^. The P123 surfactant was removed by a Soxhlet apparatus using acidic ethanol. The final material was called Fe_3_O_4_@SiO_2_@IL-PMO.

### Preparation of Fe_3_O_4_@SiO_2_@IL-PMO/Pd

For this, 0.25 g of Fe_3_O_4_@SiO_2_@IL-PMO was completely dispersed in 40 mL of dimethyl sulfoxide (DMSO) under ultrasonic irradiation for 20 min. Then, 0.025 g of Pd (OAc)_2_·4H_2_O was added and the obtained mixture was stirred at 25 °C for 24 h. Next, the product was separated using a magnet, washed, dried at 70 °C and called Fe_3_O_4_@SiO_2_@IL-PMO/Pd.

### Procedure for Heck coupling using Fe_3_O_4_@SiO_2_@IL-PMO/Pd nanocatalyst

For this purpose, 0.48 mol% of Fe_3_O_4_@SiO_2_@IL-PMO/Pd was added to a DMF solution of Ar-X (1 mmol), alkyl acrylate (2 mmol) and base (2 mmol). This was stirred at 105 °C. After completion of the reaction, ethyl acetate (10 mL) and water (10 mL) were added and the catalyst was separated by a magnet. The mixture was decanted and the organic phase was separated and dried over Na_2_SO_4_. The desired products were obtained after evaporation of solvent and/or recrystallization.

## Results and discussion

**Figure 1:**
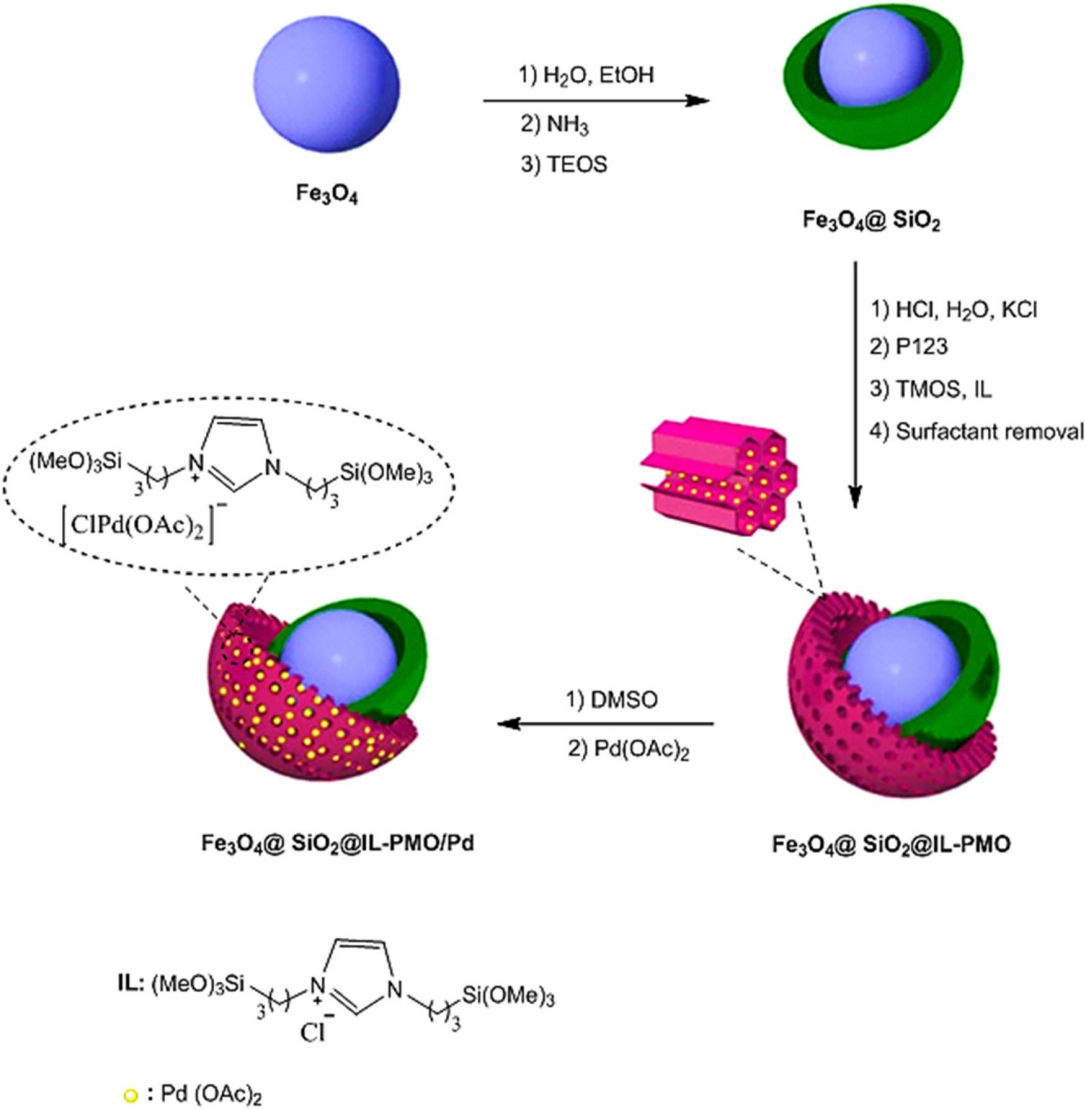
Preparation of Fe_3_O_4_@SiO_2_@IL-PMO/Pd.

The Fe_3_O_4_@SiO_2_@IL-PMO/Pd nanocomposite was prepared according to Fig. 1. As shown, Fe_3_O_4_@SiO_2_ was first prepared by coating a silica layer over the Fe_3_O_4_ surface. Then, the IL-PMO shell was created over Fe_3_O_4_@SiO_2_ via hydrolysis and co-condensation of TMOS and ionic liquid in the presence of pluronic p123 template. The Fe_3_O_4_@SiO_2_@IL-PMO/Pd nanocomposite was finally obtained via treatment of Fe_3_O_4_@SiO_2_@IL-PMO with Pd(OAc)_2_.

**Figure 2:**
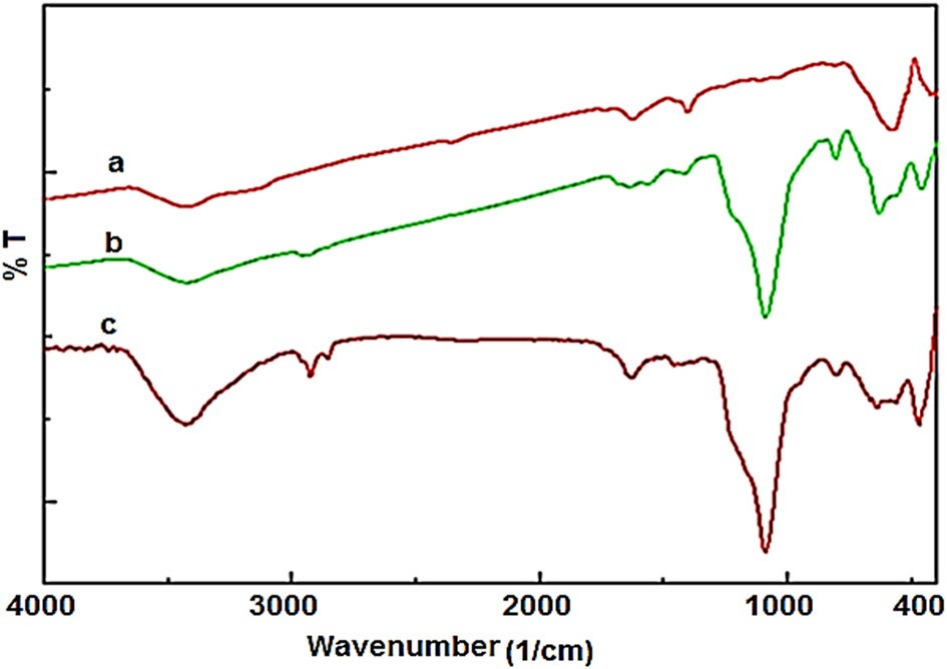
FT-IR spectra of (**a**) Fe_3_O_4_, (**b**) Fe_3_O_4_@SiO_2_ and (**c**) Fe_3_O_4_@SiO_2_@IL-PMO/Pd materials.

Figure 2 shows the FT-IR spectra of prepared materials. For all samples, the bands appeared at 586 and 3400 cm^-1^ are, respectively, assigned to Fe–O and O–H bonds (Fig. 2). For the Fe_3_O_4_@SiO_2_ and Fe_3_O_4_@SiO_2_@IL-PMO/Pd materials, the peaks at 823 and 1077 cm^-1^ are assigned to Si–O–Si bands indicating successful coating of amorphous silica on Fe_3_O_4_ (Fig. 2b). Moreover, for the Fe_3_O_4_@SiO_2_@IL-PMO/Pd material, the peaks appeared at 2923, 1420, and 1625 cm^−1^ are, respectively, due to the vibrations of aliphatic C–H, C=C and C=N bands of IL rings (Fig. 2c). These results confirm the successful coating of silica and IL-based periodic mesoporous organosilica shells over magnetite NPs.

**Figure 3:**
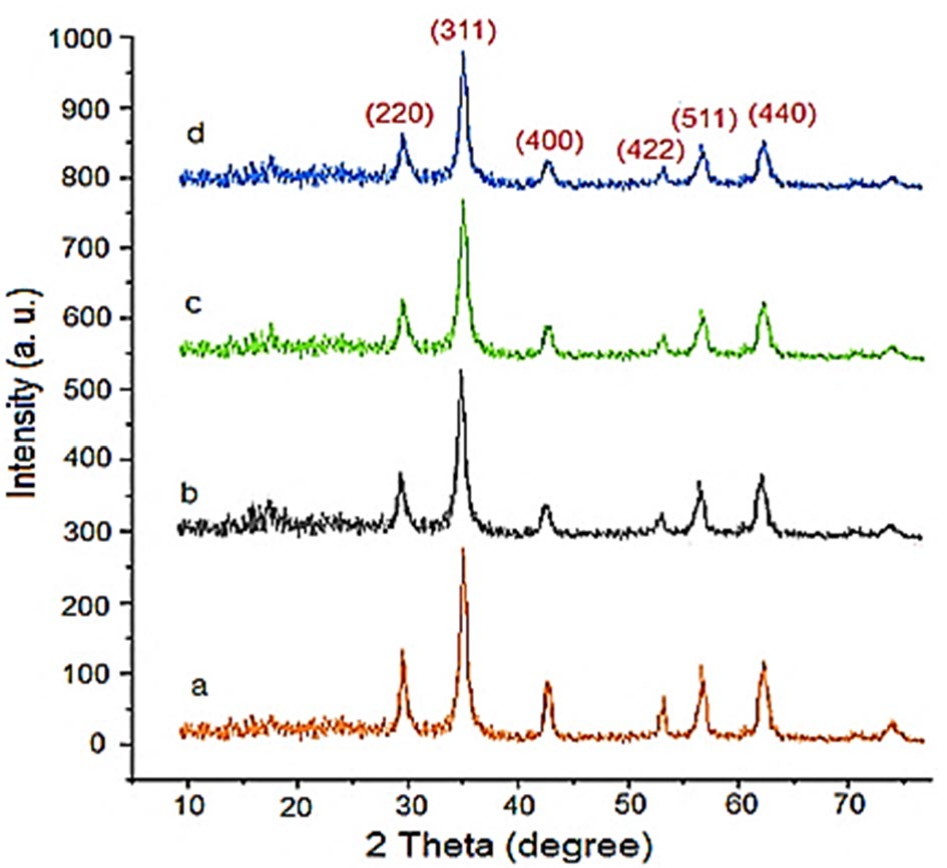
(**a**) Wide-angle PXRD of (**a**) Fe_3_O_4_, (**b**) Fe_3_O_4_@SiO_2_, (**c**) Fe_3_O_4_@SiO_2_@IL-PMO and (**d**) Fe_3_O_4_@SiO_2_@IL-PMO/Pd.

Figure 3 shows the wide-angle PXRD analysis of Fe_3_O_4_, Fe_3_O_4_@SiO_2_, Fe_3_O_4_@SiO_2_@IL-PMO and Fe_3_O_4_@SiO_2_@IL-PMO/Pd nanoparticles. The signals at 30.3, 35.7, 43.4, 53.8, 57.7 and 63.0 are, respectively, due to the reflections of 220, 311, 400, 422, 511 and 440. This confirms high stability of crystalline structure of magnetite NPs during catalyst preparation. It is also important to note that, for Fe_3_O_4_@SiO_2_, Fe_3_O_4_@SiO_2_@IL-PMO and Fe_3_O_4_@SiO_2_@IL-PMO/Pd materials, the intensity of PXRD peaks is decreased, indicating the successful modification of magnetite NPs with SiO_2_, IL-PMO and palladium moieties.

**Figure 4:**
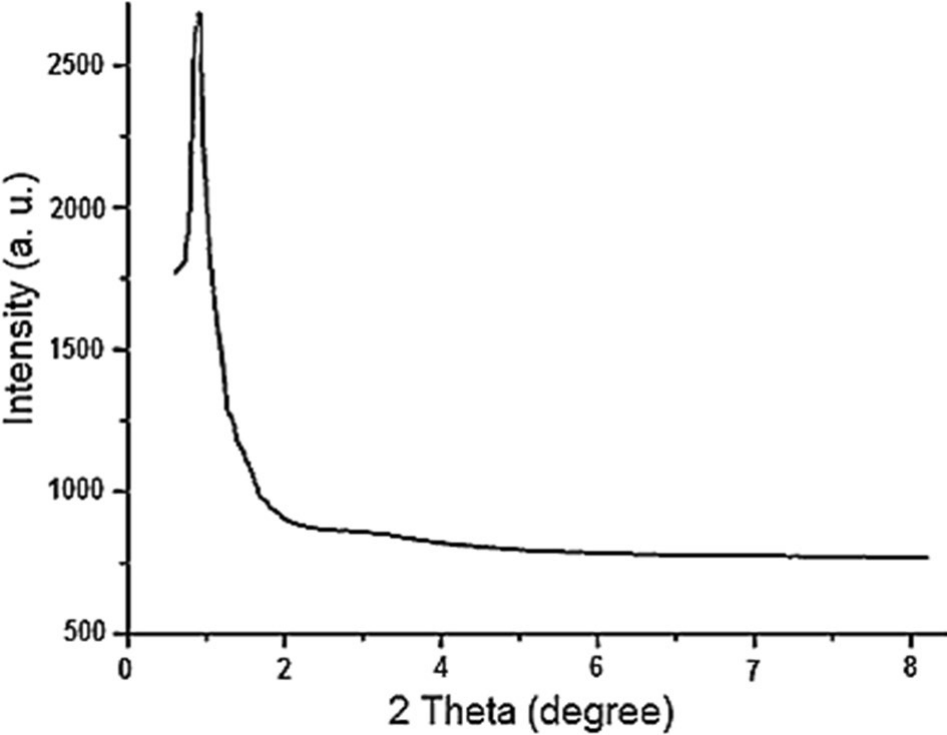
Low-angle PXRD patterns of Fe_3_O_4_@SiO_2_@IL-PMO/Pd.

The low-angle PXRD analysis of the Fe_3_O_4_@SiO_2_@IL-PMO/Pd nanocomposite demonstrated a sharp peak at 2θ≈1 corresponding to the IL-PMO shell (Fig. 4).

**Figure 5:**
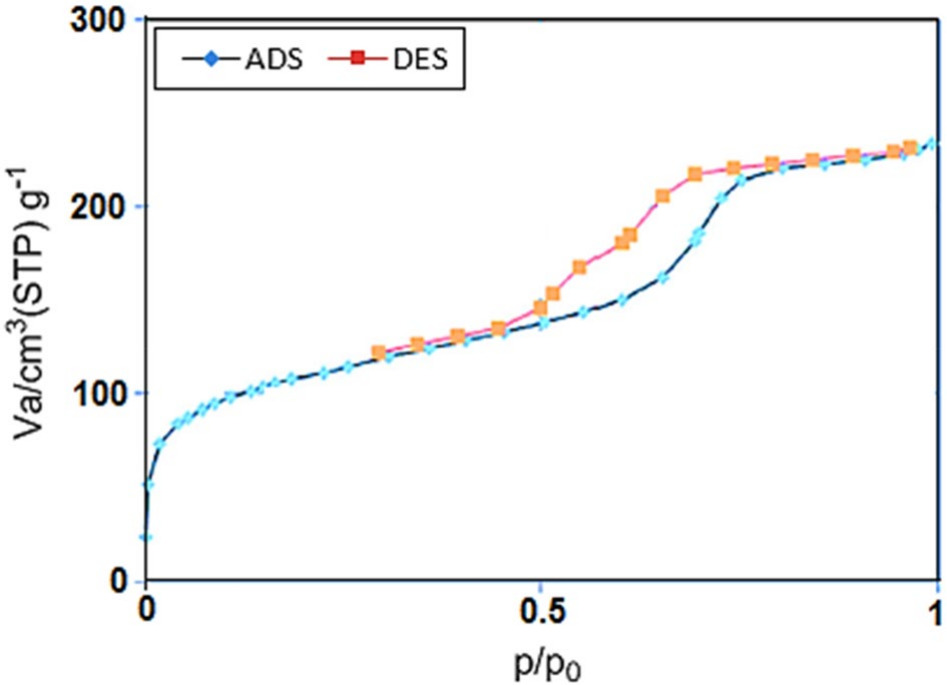
N_2_ adsorption–desorption isotherm of Fe_3_O_4_@SiO_2_@IL-PMO/Pd.

The N_2_ adsorption–desorption isotherm of the Fe_3_O_4_@SiO_2_@IL-PMO/Pd showed a type IV isotherm with a H1 hysteresis loop, which is characteristic of ordered mesostructures with high regularity (Fig. 5). Also, the BET surface area, average pore size and total pore volume of the designed Fe_3_O_4_@SiO_2_@IL-PMO/Pd nanocomposite were found to be 496.29 m^2^/g, 4.64 nm and 0.76 cm^3^/g, respectively. These results are in good agreement with low-angle PXRD analysis proving well formation of an ordered PMO shell for Fe_3_O_4_@SiO_2_@IL-PMO/Pd.

**Figure 6:**
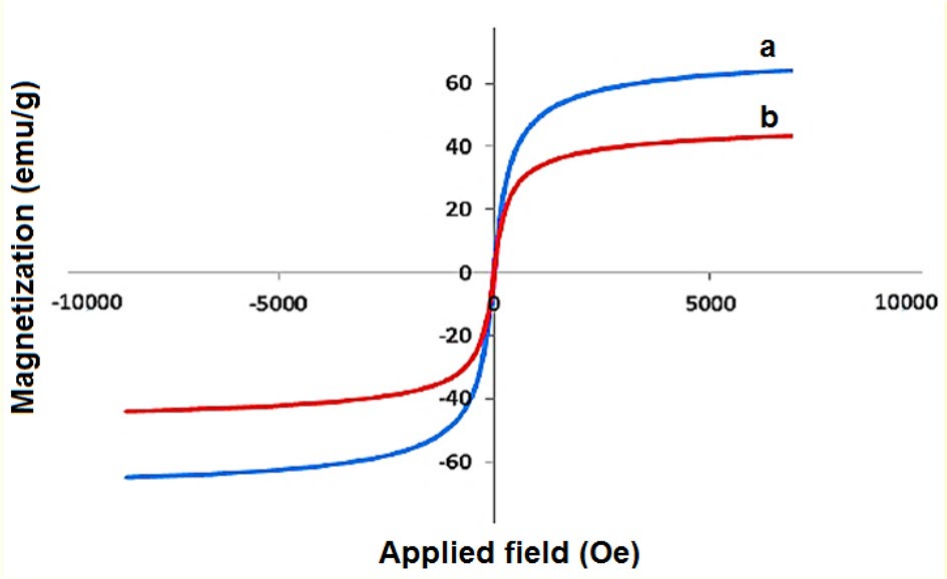
VSM analysis of the (**a**) Fe_3_O_4_ and (**b**) Fe_3_O_4_@ SiO_2_@IL-PMO/Pd.

The VSM analysis was performed to investigate the magnetic properties of Fe_3_O_4_@SiO_2_@IL-PMO/Pd (Fig. 6). This showed a saturation magnetization about 45 emu·g^−1^, which is lower than that of pure magnetic iron oxide NPs (60 emu g^−1^)^44^. This proves the successful coating of SiO_2_ and PMO shells over magnetite NPs and also confirms the high magnetic properties of the catalyst which is an excellent characteristic in the catalytic fields.

**Figure 7:**
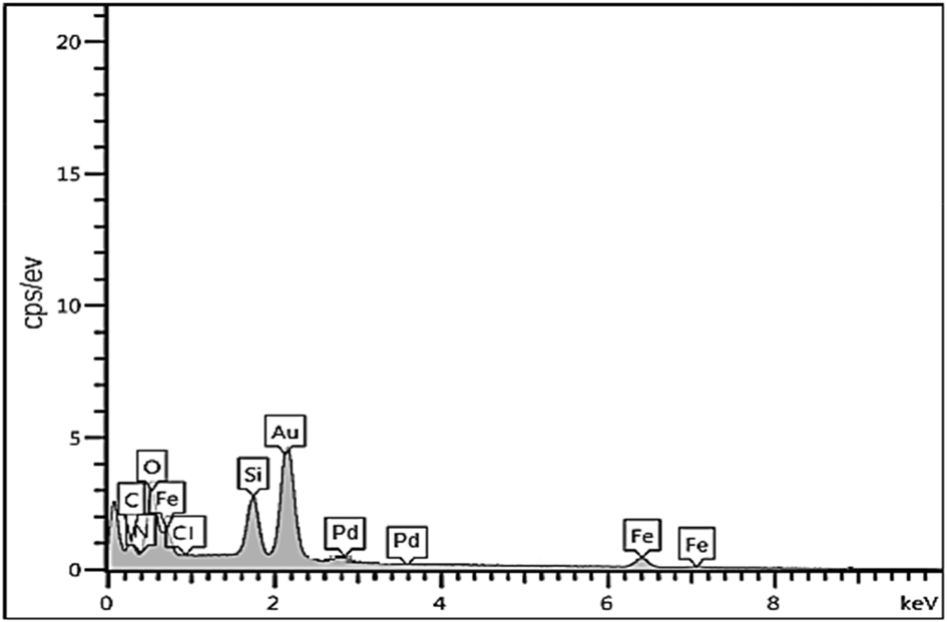
EDX analysis of Fe_3_O_4_@SiO_2_@IL-PMO/Pd.

The EDX pattern of Fe_3_O_4_@SiO_2_@IL-PMO/Pd demonstrated the signals of Fe, O, Si, C, Cl, Pd and N elements, conforming successful coating/immobilization of SiO_2_, ionic liquid and Pd moieties on magnetite NPs (Fig. 7).

**Figure 8:**
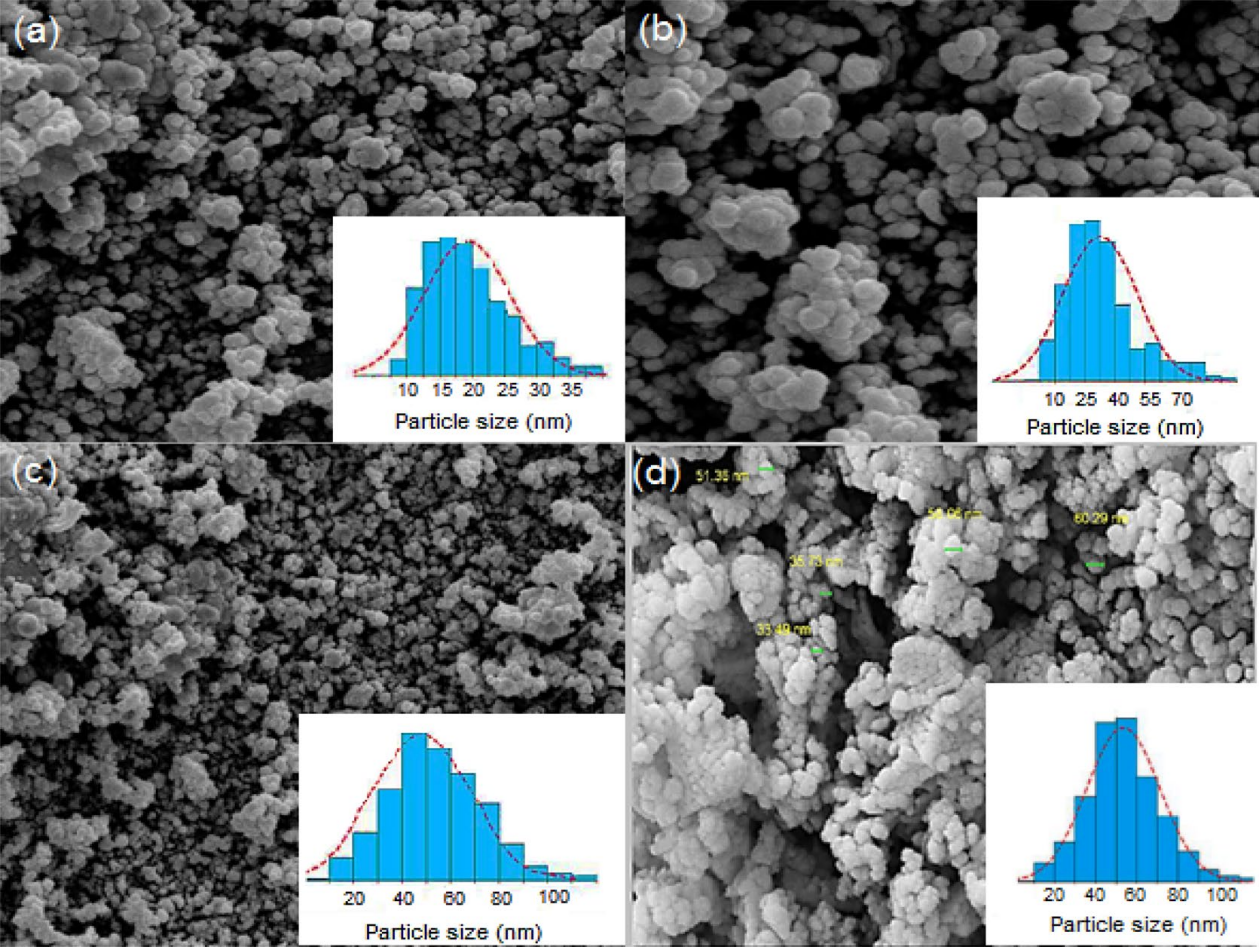
SEM image of (**a**) Fe_3_O_4_, (**b**) Fe_3_O_4_@SiO_2_, (**c**) Fe_3_O_4_@SiO_2_@IL-PMO and (**d**) Fe_3_O_4_@SiO_2_@IL-PMO/Pd materials.

**Figure 9:**
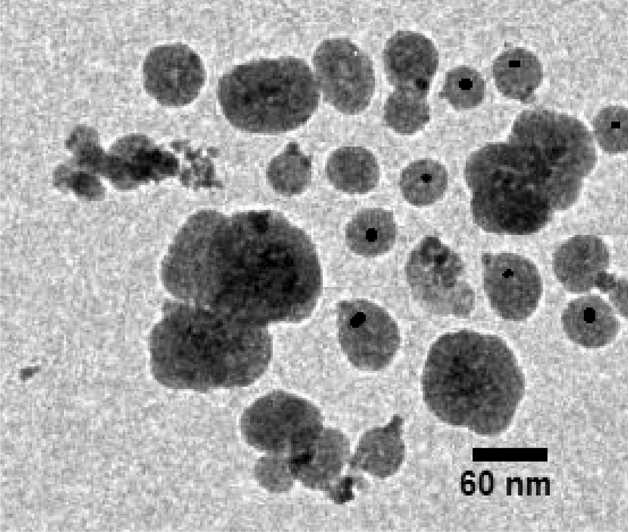
TEM image of Fe_3_O_4_@SiO_2_@IL-PMO/Pd.

The SEM analysis of Fe_3_O_4_, Fe_3_O_4_@SiO_2_, Fe_3_O_4_@SiO_2_@IL-PMO and Fe_3_O_4_@SiO_2_@IL-PMO/Pd showed a uniform spherical morphology for all samples (Fig. 8). Furthermore, according to the histogram of the SEM images (Fig. 9, inset), the average particle size of Fe_3_O_4_, Fe_3_O_4_@SiO_2_, Fe_3_O_4_@SiO_2_@IL-PMO and Fe_3_O_4_@SiO_2_@IL-PMO/Pd NPs were 20.00 ± 2.10, 30.11 ± 2.12, 49.20 ± 2.30 and 51.22 ± 2.42 nm, respectively. The increase in the particle size after each step confirms the successful shell formation and modification of magnetite NPs according to Fig. 1.

The TEM image of Fe_3_O_4_@SiO_2_@IL-PMO/Pd material also showed spherical particles with a black core (magnetite NPs) and gray shell (SiO_2_@IL-PMO layer) (Fig. 9).

**Figure 10:**
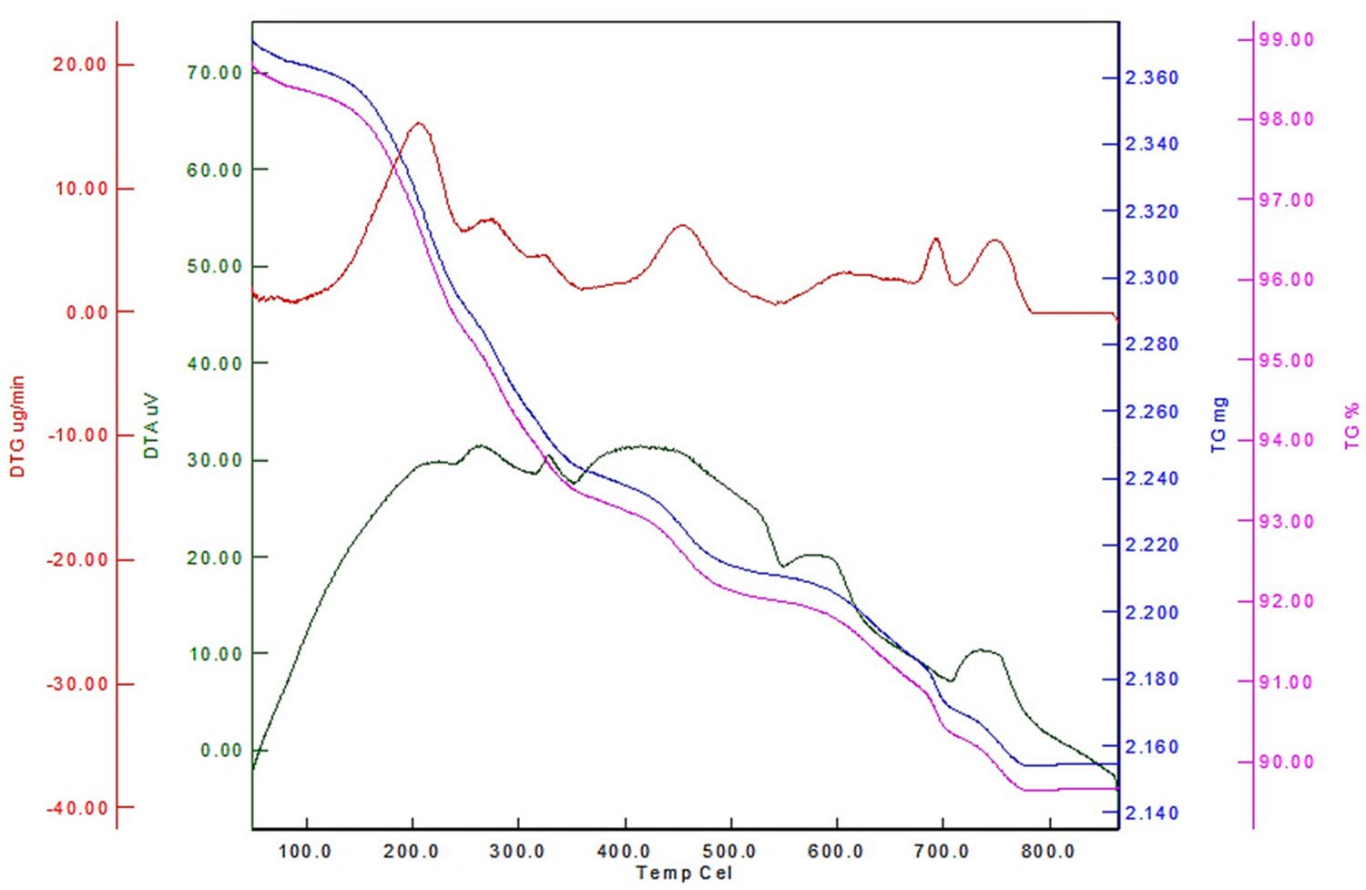
The TG analysis of Fe_3_O_4_@SiO_2_@IL-PMO/Pd.

According to TG analysis, a weight loss of about 9% was observed corresponding to the immobilized/incorporated ionic liquid groups onto/into material framework (Fig. 10).

The Heck reaction was selected as a valuable coupling reaction to evaluate the catalytic activity of Fe_3_O_4_@SiO_2_@IL-PMO/Pd as a heterogeneous catalyst. The Heck reaction between iodobenzene and ethyl acrylate was selected as a test model. The effect of solvent showed that DMF is the best giving an excellent yield of 98% (Table 1, entries 1–5). The study also showed that the rate of reaction is affected by the amount of the catalyst. As shown, the reaction yield is increased with increasing catalyst loading from 0.24 to 0.48 mol% (Table 1, entry 5 *vs* entry 6). Among various bases, K_2_CO_3_ was the most effective compared to others (Table 1, entry 5 *vs* entries 8–11). Screening different temperatures showed that at 105 °C the best result is delivered (Table 1, entry 5 *vs* entries 12, 13). Accordingly, the use of 0.48 mol% of Fe_3_O_4_@SiO_2_@IL-PMO/Pd and DMF at 105 ºC were selected as optimum conditions. In the next study, this Heck reaction was performed using Pd-free Fe_3_O_4_@SiO_2_@IL-PMO and Fe_3_O_4_@SiO_2_ materials under the same conditions as Fe_3_O_4_@SiO_2_@IL-PMO/Pd. Interestingly, in the latter study no conversion was observed indicating that the process is actually catalyzed by supported Pd species (Table 1, entry 5 *vs* entries 14, 15).

**Table 1:** Screening different parameters in the Heck reaction.


Entry	Solvent	Base	Catalyst (mol%)	T (ºC)	Yield (%)
1	EtOH	K_2_CO_3_	0.48	105	35
2	CH3CN	K_2_CO_3_	0.48	105	60
3	Toluene	K_2_CO_3_	0.48	105	65
4	NMP	K_2_CO_3_	0.48	105	85
5	DMF	K_2_CO_3_	0.48	105	98
6	DMF	K_2_CO_3_	0.24	105	85
7	DMF	K_2_CO_3_	0.97	105	98
8	DMF	NEt_3_	0.48	105	88
9	DMF	K_3_PO_4_	0.48	105	60
10	DMF	NaOAc	0.48	105	76
11	DMF	NaOH	0.48	105	55
12	DMF	K_2_CO_3_	0.48	85	20
13	DMF	K_2_CO_3_	0.48	120	98
14	DMF	K_2_CO_3_	Fe_3_O_4_@SiO_2_@ IL-PMO (0.004 g)	105	N. R.
15	DMF	K_2_CO_3_	Fe_3_O_4_@ SiO_2_ (0.004 g)	105	N. R.

After optimization, the catalyst was employed in the Heck-coupling reaction for the preparation of some styrene derivatives. As shown in Table 2, all aryl halides bearing both electron-withdrawing and electron-donating substituents reacted effectively with acrylates to give corresponding Heck products in high yield. This demonstrates high efficiency of Fe_3_O_4_@SiO_2_@IL-PMO/Pd nanocomposite for the preparation of a wide-range of important arylalkenes.

**Table 2:** Heck reaction of aryl halides and acrylates using Fe_3_O_4_@SiO_2_@IL-PMO/Pd catalyst.


Entry	X	R_1_	R_2_	Time	Yield (%)
1	I	H	CO_2_Et	30 min	98
2	I	H	CO_2_Me	30 min	98
3	I	H	CO_2_Bu	40 min	96
4	Br	H	CO_2_Et	2 h	95
5	Br	H	CO_2_Me	2 h	97
6	Br	H	CO_2_Bu	2.5 h	95
7	Br	4-MeO	CO_2_Me	10 h	90
8	Cl	4-MeO	CO_2_Me	17 h	85
9	Br	4-NO_2_	CO_2_Me	7 h	96
10	Cl	4-CHO	CO_2_Bu	12 h	89

**Figure 11:**
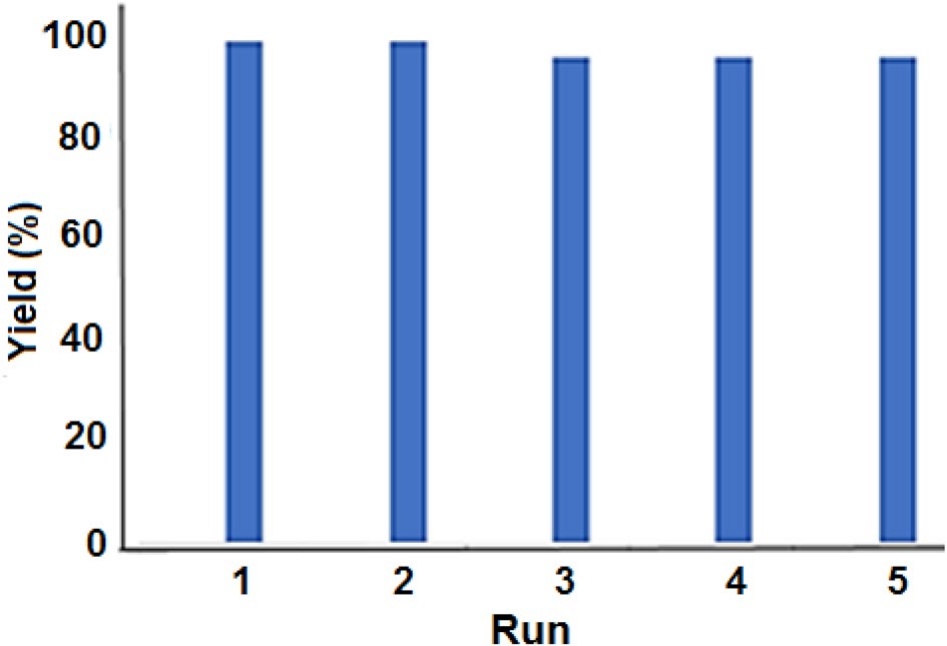
The recovery of Fe_3_O_4_@SiO_2_@IL-PMO/Pd.

The recovery of Fe_3_O_4_@SiO_2_@IL-PMO/Pd was also investigated under optimum conditions. For this, after each reaction cycle, the catalyst was removed magnetically and after washing and drying, it was reused in the next run. The results showed that the catalyst could be recovered and reused for four times with no important reduction in its performance (Fig. 11).

## Conclusion

In this study, a novel core–shell structured Fe_3_O_4_@SiO_2_@IL-PMO/Pd nanocomposite was synthesized and characterized. The well immobilization/incorporation and high stability of ionic liquid and palladium moieties over magnetite NPs were confirmed by FT-IR, TG and EDX analyses. The VSM and PXRD showed good magnetic properties of Fe_3_O_4_@SiO_2_@IL-PMO/Pd. The nitrogen-sorption and low-angle PXRD showed a mesoporous shell for the designed material. This nanocomposite was catalytically employed in the Heck reaction giving high yield of corresponding coupling products. The recovery test demonstrated high stability and durability of active catalytic species during applied conditions.
